# Effectiveness of dasabuvir/ombitasvir/paritaprevir/ritonavir for hepatitis C virus in clinical practice: A population-based observational study

**DOI:** 10.1371/journal.pone.0176858

**Published:** 2017-07-07

**Authors:** Maya Leventer-Roberts, Ariel Hammerman, Ilan Brufman, Moshe Hoshen, Marius Braun, Yaffa Ashur, Nicky Lieberman, Ran Balicer

**Affiliations:** 1 Clalit Research Institute, Tel Aviv, Israel; 2 Icahn School of Medicine at Mount Sinai, New York, New York, United States of America; 3 Chief Physician's Office, Clalit Health Services, Tel Aviv, Israel; 4 Liver Unit, Beilinson Hospital, Petach Tikva, Israel; 5 Sackler School of Medicine, Tel Aviv University, Tel Aviv, Israel; 6 Hepatology, Clalit Health Services, Tel Aviv, Israel; 7 Community Medicine Division, Clalit Health Services, Tel Aviv, Israel; 8 Department of Epidemiology, Faculty of Health Sciences, Ben Gurion University, Be'er Sheva, Israel; University of North Carolina at Chapel Hill School of Dentistry, UNITED STATES

## Abstract

**Background:**

Direct acting antivirals for hepatitis C virus have shown dramatic results in clinical trials. However, their effectiveness has yet to be demonstrated within observational cohorts which lack exclusion criteria found in randomized control trials.

**Aim:**

To determine the effectiveness of dasabuvir/ombitasvir/paritaprevir/ritonavir in achieving sustained virological response.

**Methods:**

Retrospective observational cohort study of all Clalit Health Services members with hepatitis C virus genotype 1 who were dispensed dasabuvir/ombitasvir/paritaprevir/ritonavir from January 1, 2015 to-November 31, 2015.

**Results:**

There were 564 participants during the study period. The average age was 61.9 years, 52.0% were male, and 61.5% were born Eastern/Central Europe or Central Asia. The prevalence of diabetes was 31.7% and 70.3% were overweight/obese. Cirrhosis was present in 41.0% of participants, of whom 52.8% had stage 4 fibrosis. Of the cohort, 416 (74.8%) had follow-up viral load testing at 10 or more weeks after the end of treatment. We report a sustained virological response of 98.8% among those tested.

**Conclusions:**

Treatment with dasabuvir/ombitasvir/paritaprevir/ritonavir demonstrated a near universal effectiveness in achieving a sustained virological response among HCV patients in a large cohort.

## Introduction

In recent clinical trials, direct acting antivirals (DAAs) for chronic infection with hepatitis C virus (HCV) have demonstrated very high efficacy rates, in some cases >95% in achieving sustained virological responses among participants with low grade fibrosis [1–8]. Without such treatments, chronic infection with HCV has a major impact on quality of life and healthcare utilization [9, 10], including long-term complications such as liver failure, the need for liver transplant, the development of hepatic cancer, and an elevated risk for mortality [11].

However, these trials have usually selected participants who do not necessarily represent all those infected with HCV, as some trials excluded participants who were previously treated for HCV with any antiviral therapy, specifically DAAs, or who had evidence of cirrhosis [1, 2, 5, 12]. While several observational studies [5, 6, 13–16] have studied DAA treatment effectiveness among cohorts with cirrhosis and human immunodeficiency virus (HIV) or through the United States Veterans Administration [13, 17], no study has evaluated the effectiveness among a diverse group of participants from a general population as large as that available in Clalit Health Services (CHS).

The aim of this study was to assess the effectiveness of the dasabuvir/ombitasvir/paritaprevir/ritonavir (3D) protocol in achieving a sustained virological response (SVR) among a diverse group of participants with HCV.

## Materials and methods

### Design overview

This was a retrospective observational cohort study comprised of members enrolled in CHS, Israel’s largest integrated payer-provider system, who were dispensed at least one prescription of the 3D protocol after January 1, 2015, and prior to November 30, 2015 (allowing for sufficient follow-up as of the date of data extraction on February 21, 2016).

The study was approved by CHS’s institutional review board.

### Setting

Healthcare in Israel is mandatory and universal. All citizens and residents can freely choose membership in any of the four integrated payer-provider healthcare systems, which are required by law to offer the same basic list of services, including prescription medications on the National List of Reimbursed Drugs. CHS’s membership is comprised of over half of the Israeli population (more than 4.3 million people) and the switching rate between providers is low (less than 2% annually) [18].

In Israel, the 3D protocol was the first new DAA protocol to be included in the National List of Reimbursed Drugs and became available in January 2015 for all people infected with HCV (genotype 1a or 1b) who had concurrent advanced liver disease (fibrosis stage 3 or 4). CHS introduced an additional recommendation for participants to undergo a viral load test in the first month following treatment initiation to assess treatment responsiveness.

### Data sources

CHS's fully integrated electronic health records database was accessed for this study. These records include members’ demographic characteristics and clinical co-variates (outpatient and inpatient), laboratory values, medication prescription and purchasing information, as well as clinical markers such as body mass index (BMI) and patient-reported data. The data are linked according to each member’s unique national identification number which is used anonymously in research studies.

### Case definition

All CHS members who were dispensed at least one prescription for the 3D protocol during the study period were included in the study. This study had no further inclusion or exclusion criteria.

### Exposure

Participants who were prescribed the 3D protocol received a single tablet of dasabuvir 250 mg to be taken twice daily, and two tablets containing a combination of ombitasvir 12.5 mg, paritaprevir 75 mg, and ritonavir 50 mg to be taken once daily. Genotype 1b with compensated cirrhosis and genotype 1a without cirrhosis or with compensated cirrhosis also received ribavirin. The suggested treatment duration was a 24-week course for participants with HCV genotype 1a and compensated cirrhosis, and all others were to receive a 12-week course. However, there was allowance for clinical judgment in choosing which treatment duration to prescribe.

### Main outcome measures

The primary outcome of this study was a sustained virological response (SVR) or a non-detectable viral load (<15 IU/mL) at 10 or more weeks after the end of treatment. The 10-week duration was chosen due to the potential variation in local community clinic practices and laboratory processing, as cited previously in similar effectiveness studies [11, 13, 17]. Three additional viral load test results are provided for comparison: the baseline viral load test in IU/mL prior to initiation of treatment (<15, ≥15 to <800,000, ≥800,000 to <2 million, ≥2 million to <6 million, ≥6 million, and positive, non-quantifiable), an early response assessed at 4 weeks (+/- 2 weeks) following the initiation of treatment, and a viral load at the end of treatment (+/- 2 weeks), as determined by the date of the first purchase and the duration of the approved regimen. These latter values are provided categorically in IU/mL (≥15 to <1,000, ≥1,000 to <1 million, ≥1 million).

The 3D protocol was only dispensed as a complete 4-week supply. Ribavirin, when prescribed, could be dispensed in any number. Adherence is calculated as the proportion of days covered. Adherence of 80% or more to the treatment regimen was considered adherent.

### Demographic characteristics and co-morbidities

Demographic characteristics on members included age, sex, region of birth [19], and socioeconomic status (as defined by the Israeli Central Bureau of Statistics). Clinical co-variates considered in the study included: diabetes (identified using an algorithm previously-validated within the Clalit system [20]), chronic kidney disease (CKD) (staging based on estimated glomerular filtration rate [eGFR] calculated using CKD-Epidemiology Collaboration [EPI]: stage 1, stage 2, stage 3a, stage 3b, stage 4, stage 5, renal replacement therapy (any documentation of end stage renal disease, kidney failure, dialysis, or renal transplant), Charlson morbidity score [21], morbidity burden based on the resource utilization bands of the Adjusted Clinical Groups^®^ (ACG) system [22] (containing 5 groups of resource utilization; 1 represents the lowest burden and 5 represents the highest burden), BMI category (kg/m2: underweight ≤18.5, normal weight 18.5 to ≤25, overweight 25 to ≤30, and obese 30 or more), and smoking status (current, former, and non-smoker).

Hepatitis-specific markers included alanine transaminase (ALT), aspartate aminotransferance (AST), platelets, AST to platelet ratio index (APRI) score, presence of cirrhosis according to the International Statistical Classification of Diseases, Ninth Revision diagnosis codes (any, compensated [cirrhosis or esophageal varices], decompensated [encephalopathy, esophageal varies and bleeding, portal syndrome, jaundice, or ascites]), stage of fibrosis (as determined by transient elastography, biomarkers, or liver biopsy), diagnosis of liver transplant, co-infection with laboratory-confirmed HIV or hepatitis B virus (HBV) (positive HBsAg), and years from first confirmed HCV diagnosis (earliest among laboratory tests for antibody, polymerase chain reaction [PCR], genotype, or viral load) were also included. Any prior treatment regimen was recorded (specifically, of at least one purchase of the following regimens: peginterferon and ribavirain; peginterferon, ribavirin, and boceprevir; peginterferon, ribavirin, and telaprevir; or peginterferon).

### Statistical analysis

Basic demographic characteristics and clinical co-variates of the participants in the study population were compared to those of the general CHS population who were in the same age range as the participants (21 to 90 years old, unadjusted). These characteristics were extracted on the date of treatment initiation for participants and on January 1, 2015 for the general CHS population. Participants were categorized by genotype sub-groups (1a, 1b, or unknown) due the known association with country of birth and the potential socioeconomic differences between those groups and the treatment duration (12-weeks or 24-weeks). Baseline hepatitis-specific markers, co-morbidities, and primary and secondary outcomes were also compared among the participants of the study. Finally, demographic characteristics, clinical co-variates, and hepatitis-specific markers were compared between participants who were and were not assessed for SVR to examine whether there were any significant differences between groups that could suggest a bias in the outcomes. Categorical comparisons were conducted using Fisher exact test for nominal comparisons and Cochran-Armitage for ordinal comparisons. Bivariate continuous variables were compared using Student t-test for normal distributions and Mann-Whitney for non-parametric tests. The statistical software used was SPSS version 22.0 (IBM, Chicago IL).

## Results

There were 564 participants who were included in the study (Table 1). The mean age was 61.8 years, 52.0% were female and 61.5% were born in the areas with high HCV prevalence (Eastern/Central Europe or Central Asia). In comparison, the average age of the general CHS population was 46.0 years and 14.2% were born in Eastern/Central Europe or Central Asia.

**Table 1:** Demographic characteristics of all CHS members and participants, by assigned treatment duration and HCV genotype*.

	All CHS Members (n = 2,746,913)	Total Participants by treatment duration			All CHS vs Total Participants P value	HCV Genotype 1a by treatment duration			HCV Genotype 1b by treatment duration			HCV Genotype 1a vs HCV Genotype 1b P value	HCV Genotype 1 Subtype Unknown by treatment duration		
		12-week (n = 494)	24-week (n = 70)	All (n = 564)		12-week (n = 32)	24-week (n = 54)	All (n = 86)	12-week (n = 430)	24-week (n = 9)	All (n = 439)		12-week (n = 32)	24-week (n = 7)	All (n = 39)
**Demographic Characteristics**															
Age, y, mean (SD)	46 (18.01)	61.94 (11.69)	60.59 (9.73)	61.77 (11.47)	<.001	57.88 (8.10)	59.19 (9.46)	58.70 (8.95)	62.11 (12.07)	64.56 (9.28)	62.16 (12.01)	.002	63.66 (8.54)	66.29 (10.19)	64.13 (8.78)
Age groups, y															
<55	1,793,399 (65)	106	15	121 (22)		10	14	24 (28)	93	1	94 (21)		3	0	3 (8)
55-<65	412,068 (15)	165	35	200 (36)		17	29	46 (53)	129	3	132 (30)		19	3	22 (56)
≥ 65	541,446 (20)	223	20	243 (43)		5	11	16 (19)	208	5	213 (49)		10	4	14 (36)
Sex					NS							.005			
Male	1,318,131 (48)	249	44	293 (52)	0.85 (0.72–1.01)^†^	21	36	57 (66)	212	5	217 (49)	2.01 (1.24–3.26)^†^	16	3	19 (49)
Region of Birth by WHO GBD															
Eastern/Central Europe and Central Asia	390,770 (14)	334	13	347 (62)		9	6	15 (17)	310	4	314 (72)		15	3	18 (46)
Western Europe/Israel	1,965,389 (72)	94	42	136 (24)	<.001	17	36	53 (62)	65	3	68 (16)	<.001	12	3	15 (39)
North Africa/Middle East	257,351 (9)	59	15	74 (13)		6	12	18 (21)	48	2	50 (11)		5	1	6 (15)
Other/Unknown	133,403 (5)	7	0	7 (1)		0	0	0 (0)	7	0	7 (2)		0	0	0 (0)
SES															
Low	1,136,143 (41)	183	19	202 (36)		17	14	31 (36)	157	2	159 (36)		9	3	12 (31)
Medium	1,033,580 (38)	224	36	260 (46)	<.001	10	31	41 (48)	197	3	200 (46)	NS	17	2	19 (49)
High	571,428 (21)	87	15	102 (18)		5	9	14 (16)	76	4	80 (18)		6	2	8 (21)
Missing data	5,762 (0)	0	0	0 (0)		0	0	0 (0)	0	0	0 (0)		0	0	0 (0)

CHS, Clalit health services; HCV, hepatitis C virus; SD, standard deviation; y, years; WHO, World Health Organization; GBD, global burden of disease; SES, socioeconomic status; OR, Odds Ratio; 95% CI, 95% confidence interval.

* Values are numbers (percentages) unless otherwise indicated.

^†^ Values are OR (95% CI).

There were 179 study participants who had a documented diagnosis of diabetes (31.7%) as compared to the general CHS population among whom 12.8% had a diagnosis of diabetes (Table 2). The ACG category was 2 or greater for 94.0% of the participants as compared to 65.8% of the general CHS population. Among all participants, 29.1% were normal weight; in the general CHS population, 39.7% were normal weight.

**Table 2:** Clinical co-variates of all CHS members and participants, by assigned treatment duration and HCV genotype*.

	All CHS Members (n = 2,746,913)	Total Participants by treatment duration			All CHS vs Total Participants P value	HCV Genotype 1a by treatment duration			HCV Genotype 1b by treatment duration			HCV Genotype 1a vs HCV Genotype 1b P value	HCV Genotype 1 Subtype Unknown by treatment duration		
		12-week (n = 494)	24-week (n = 70)	All (n = 564)		12-week (n = 32)	24-week (n = 54)	All (n = 86)	12-week (n = 430)	24-week (n = 9)	All (n = 439)		12-week (n = 32)	24 week (n = 7)	All (n = 39)
**Clinical co-variates**															
Diabetes	352,320 (13)	151	28	179 (32)	0.32 (0.27–0.38)^†^	2	20	22 (26)	138	7	145 (33)	0.70 (0.41–1.18)^†^	11	1	12 (31)
Duration of diabetes, y															
<10	208,651 (59)	87	20	107 (60)	0.98 (0.72–1.32)^†^	1	17	18 (82)	81	3	84 (58)	3.27 (1.05–10.14)^†^	5	0	5 (42)
≥10	143,669 (41)	64	8	72 (40)		1	3	4 (18)	57	4	61 (42)		6	1	7 (58)
CKD stage															
1	1,764,995 (64)	284	47	331 (59)		24	40	64 (74)	242	1	243 (55)		18	6	24 (62)
2	632,149 (23)	184	14	198 (35)		8	11	19 (22)	162	3	165 (38)		14	0	14 (36)
3A	87,604 (3)	17	3	20 (4)	<.001	0	0	0 (0)	17	3	20 (5)	.003	0	0	0 (0)
3B	33,685 (1)	5	5	10 (2)		0	2	2 (2)	5	2	7 (2)		0	1	1 (3)
4	9,929 (0)	3	0	3 (1)		0	0	0 (0)	3	0	3 (1)		0	0	0 (0)
5 Non- Dialysis	1,490 (0)	0	0	0 (0)		0	0	0 (0)	0	0	0 (0)		0	0	0 (0)
Renal replacement therapy	6,325 (0)	0	1	1 (0)		0	1	1 (1)	0	0	0 (0)		0	0	0 (0)
Missing data	210,736 (8)	1	0	1 (0)		0	0	0 (0)	1	0	1 (0)		0	0	0 (0)
ACG categories															
0–1	919,359 (34)	29	5	34 (6)		3	5	8 (9)	22	0	22 (5)		4	0	4 (10)
2–3	1,514,585(55)	227	30	257 (46)	<.001	18	21	39 (45)	194	4	198 (45)	NS	15	5	20 (51)
4–5	294,792 (11)	238	35	273 (48)		11	28	39 (45)	214	5	219 (50)		13	2	15 (39)
Missing data	18,177 (1)	0	0	0 (0)		0	0	0 (0)	0	0	0 (0)		0	0	0 (0)
Charlson with age															
0	1,233,878 (45)	31	5	36 (6.4)		3	5	8 (9)	27	0	27 (6)		1	0	1 (3)
1	459,117 (17)	58	10	68 (12)	<.001	6	8	14 (16)	48	0	48 (11)	NS	4	2	6 (15)
2+	998,764 (36)	388	54	442 (78)		22	40	62 (72)	340	9	349 (80)		26	5	31 (80)
Missing data	55,154 (2)	17	1	18 (3)		1	1	2 (2)	15	0	15 (3)		1	0	1 (3)
BMI															
Underweight	81,930 (3)	3	1	4 (1)		1	1	2 (2)	2	0	2 (1)		0	0	0 (0)
Normal weight	1,090,308 (40)	143	21	164 (29)		15	16	31 (36)	121	4	125 (29)		7	1	8 (21)
Overweight	909,866 (33)	197	34	231 (41)	<.001	11	26	37 (43)	176	3	179 (41)	.013	10	5	15 (39)
Obese	592,670 (22)	151	14	165 (29)		5	11	16 (19)	131	2	133 (30)		15	1	16 (41)
Missing data	72,139 (3)	0	0	0		0	0	0 (0)	0	0	0 (0)		0	0	0 (0)
Smoking															
Non-smoker	1,763,061 (64)	293	26	319 (57)		8	16	24 (28)	264	6	270 (62)		21	4	25 (64)
Former	399,390 (15)	106	13	119 (21)	<.001	6	10	16 (19)	93	2	95 (22)	<.001	7	1	8 (21)
Current	543,429 (20)	95	31	126 (22)		18	28	46 (54)	73	1	74 (17)		4	2	6 (15)
Missing data	41,033 (1)	0	0	0		0	0	0 (0)	0	0	0 (0)		0	0	0 (0)

CHS, Clalit health services; HCV, hepatitis C virus; y, years; CKD, chronic kidney disease; ACG, adjusted clinical groups; BMI, body mass index; OR, Odds Ratio; 95% CI, 95% confidence interval.

* Values are numbers (percentages) unless otherwise indicated.

^†^ Values are OR (95% CI).

The baseline liver functions tests for all participants included the following: median ALT of 65 U/L (range 42–101 U/L), median AST of 65 U/L (range 46–96 U/L), mean platelets of 150 10^9^/L (range 106–207 10^9^/L), and median APRI score of 1.35 (range 0.73–2.37) (Table 3). Overall, 52.8% of participants had stage 4 fibrosis and 41.0% had cirrhosis. In total, 10 participants were co-infected with HIV and 6 co-infected with HBV. Among those with genotype 1a, 90.7% had had any type of prior treatment regimen, and among those with genotype 1b, 67.7% had had any type of previous HCV treatment.

**Table 3:** Hepatitis-specific markers of participants, by assigned treatment duration and HCV genotype*.

	Total Participants by treatment duration			HCV Genotype 1a by treatment duration			HCV Genotype 1b by treatment duration			HCV Genotype 1a vs HCV Genotype 1b P value	HCV Genotype 1 Subtype Unknown by treatment duration		
	12-week (n = 494)	24-week (n = 70)	All (n = 564)	12-week (n = 32)	24-week (n = 54)	All (n = 86)	12-week (n = 430)	24-week (n = 9)	All (n = 439)		12-week (n = 32)	24-week (n = 7)	All (n = 39)
**Hepatitis-specific markers**													
ALT, U/L													
mean (SD)	82.32 (64.67)	74.42 (44.32)	81.33 (62.52)	69.6 3 (41.21)	76.55 (41.77)	73.97 (41.46)	83.10 (65.72)	58.22 (58.94)	82.59 (65.62)	NS	84.53 (69.74)	78.86 (45.69)	83.5 1 (65.59)
median (range)	65.00 (42.50–101.00)	63.00 (37.75–101.50)	65.00 (42.00–101.00)	53.50 (37.75–93.50)	71.25 (43.25–101.50)	69.50 (40.00–96.00)	65.00 (42.50–102.00)	30.00 (26.00–88.00)	64.50 (42.00–102.00)	NS	70.50 (49.50–86.25)	62.00 (53.00–119.00)	68.00 (51.00–87.00)
AST, U/L													
mean (SD)	78.93 (49.12)	75.18 (38.87)	78.46 (47.95)	62.97 (32.21)	77.95 (38.45)	72.38 (36.79)	79.92 (47.39)	56.89 (44.00)	79.45 (47.39)	NS	81.56 (77.19)	78.86 (45.69)	80.79 (71.04)
median (range)	64.00 (46.00–98.00)	71.00 (51.75–87.00)	65.00 (46.00–96.00)	61.50 (37.25–79.75)	74.50 (53.50–88.75)	71.00 (49.00–87.00)	66.00 (46.00–102.00)	55.00 (26.50–66.50)	65.50 (46.00–101.25)	NS	58.00 (50.00–80.25)	66.00 (64.00–117.00)	62.00 (50.00–83.00)
Platelets, 10^9^/L													
mean (SD)	162.35 (75.48)	142.09 (68.85)	159.83 (74.93)	191.44 (65.44)	145.13 (71.65)	162.36 (72.60)	159.48 (76.37)	119 (50.25)	158.65 (76.10)	NS	171.81 (67.37)	148.29 (69.77)	167.59 (67.49)
median (range)	152.50 (109.00–208.00)	125.00 (90.75–204.50)	150.00 (106.00–207.00)	176.50 (147.00–236.25)	131.00 (89.50–209.50)	161.00 (104.25–215.25)	150.50 (105.75–204.00)	110.00 (87.50–161.00)	149.00 (105.00–203.00)	NS	180.00 (115.75–227.00)	108.00 (100.00–247.00)	146.00 (108.00–228.00)
APRI score													
mean (SD)	2.07 (1.30)	2.04 (1.85)	2.07 (2.79)	1.28 (1.26)	2.14 (2.00)	1.82 (1.80)	2.14 (2.98)	1.62 (1.39)	2.13 (2.96)	NS	1.88 (2.82)	1.85 (0.90)	1.87 (2.57)
median (range)	1.30 (0.72–2.30)	1.67 (0.91–2.53)	1.35 (0.73–2.37)	0.82 (0.54–1.50)	1.68 (0.91–2.67)	1.32 (0.69–2.29)	1.38 (0.73–2.42)	1.21 (0.65–1.99)	1.37 (0.73–2.42)	NS	0.96 (0.76–1.73)	1.69 (1.54–2.42)	1.13 (0.78–1.88)
Fibrosis stage													
1	1	0	1 (0)	0	0	0 (0)	1	0	1 (0)		0	0	0 (0)
2	0	0	0 (0)	0	0	0 (0)	0	0	0 (0)		0	0	0 (0)
3	161	5	166 (29)	27	0	27 (31)	130	5	135 (31)	N/A	4	0	4 (10)
4	244	54	298 (53)	4	46	50 (58)	239	3	242 (55)		1	5	6 (15)
Unable to determine	0	0	0 (0)	0	0	0 (0)	0	0	0 (0)		0	0	0 (0)
Missing data	88	11	99 (18)	1	8	9 (11)	60	1	61 (14)		27	2	29 (74)
Cirrhosis													
Any	196	35	231 (41)	5	25	30 (35)	179	8	187 (43)	0.72 (0.45–1.17)^†^	12	2	14 (36)
Compensated	144	22	166 (72)	3	18	21 (70)	134	3	137 (73)	0.85 (0.37–1.98) ^†^	7	1	8 (57)
Decompensated	52	13	65 (28)	2	7	9 (30)	45	5	50 (27)		5	1	6 (43)
Liver transplant	1	3	4 (1)	0	0	0 (0)	1	3	4 (1)	N/A	0	0	0 (0)
HIV	10	0	10 (2)	2	0	2 (2)	7	0	7 (2)	1.47 (0.30–7.20) ^†^	1	0	1 (3)
HBV	6	1	7 (1)	0	1	1 (1)	6	0	6 (1)	0.85 (0.10–7.14)^†^	0	0	8 (57)
Any previous HCV treatment	345	63	408 (72)	30	48	78 (91)	289	8	297 (68)	4.66 (2.19–9.91)^†^	26	7	33 (85)
peginterferon + ribavirin	318	59	377 (67)	26	46	72 (84)	269	8	277 (63)		23	5	28 (72)
peginterferon + ribavirin + boceprevir	9	3	12 (2)	2	1	3 (4)	7	0	7 (2)	<0.001	0	2	2 (5)
peginterferon + ribavirin + telaprevir	13	1	14 (3)	2	1	3 (4)	10	0	10 (2)		1	0	1 (3)
peginterferon	5	0	5 (1)	0	0	0 (0)	3	0	3 (1)		2	0	2 (5)
None	149	7	156 (28)	2	6	8 (9)	141	1	142 (32)		6	0	6 (15)
HCV duration, y													
<1	41	9	50 (18)	0	8	8 (9)	39	0	39 (9)		2	1	3 (8)
1-<5	86	13	99 (38)	7	12	19 (22)	74	1	75 (17)		5	0	5 (13)
5-<10	191	21	212 (35)	12	14	26 (30)	167	4	171 (39)	<.001	12	3	15 (39)
≥10	173	26	199 (1)	13	19	32 (37)	147	4	151 (34)		13	3	16 (41)
Missing data	3	1	4 (1)	0	1	1 (1)	3	0	3 (1)		0	0	0 (0)

HCV, hepatitis C virus; ALT, alanine transaminase; SD, standard deviation; AST, aspartate aminotransferase; APRI, AST to platelet ratio index score; HIV, human immunodeficiency virus; HBV, hepatitis B virus; y, years; OR, Odds Ratio; 95% CI, 95% confidence interval.

* Values are numbers (percentages) unless otherwise indicated.

^†^ Values are OR (95% CI).

There were 554 participants who had a baseline viral load with a mean of 5.97 log_10_ IU/mL. An additional ten participants appeared to have baseline viral load testing performed at laboratories outside CHS, as their baseline results were either non-detectable or not available.

At the early response assessment, 454 participants had a viral load test performed, of which 73.8% had non-detectable viral levels. At the end of treatment, 331 participants had a viral load test performed, of whom, 97.9% had non-detectable levels. There were 416 participants who were assessed for an SVR (73.8% of the participants), and 98.8% of those assessed had non-detectable viral loads (Table 4). During the course of treatment, 91.7% of participants were adherent to at least 80% of the recommended regimen. Adherence was 76.8% among those with 24-week regimens and 93.5% among those with 12-week regimens.

**Table 4:** Outcomes of participants, assigned treatment duration and HCV genotype*.

		Total Participants by treatment duration			HCV Genotype 1a by treatment duration			HCV Genotype 1b by treatment duration			HCV Genotype 1a vs HCV Genotype 1b P value	HCV Genotype 1 Subtype Unknown by treatment duration		
		12-week (n = 488)	24-week (n = 68)	All (n = 556)	12-week (n = 32)	24-week (n = 52)	All (n = 84)	12-week (n = 424)	24-week (n = 9)	All (n = 433)		12-week (n = 32)	24-week (n = 7)	All (n = 39)
**Outcomes**														
Baseline viral load, log10 IU/mL, mean (SD)		5.95 (0.82)	6.09 (0.68)	5.97 (0.80)	6.17 (0.81)	6.11 (0.65)	6.13 (0.71)	5.91 (0.82)	5.86 (0.99)	5.91 (0.83)	.012	6.26 (0.65)	6.18 (0.43)	6.25 (0.61)
Viral load, IU/mL														
<15		1	1	2 (0)	0	1	1 (1)	1	0	1 (0)		0	0	0 (0)
≥15 and <800,000		190	22	212 (38)	7	18	25 (30)	174	3	177 (41)		9	1	10 (26)
≥800,000 and <2 million		120	15	135 (24)	8	10	18 (21)	105	3	108 (25)		7	2	9 (23)
≥2 million and <6 million		119	22	141 (25)	12	17	29 (34.5)	99	1	100 (23)	.016	8	4	12 (31)
≥6 million		48	8	56 (10)	4	6	10 (12)	36	2	38 (9)		8	0	8 (21)
Positive, non-quantifiable		10	0	10 (2)	1	0	1 (1)	9	0	9 (2)		0	0	0 (0)
Total		488	68	556 (100)	32	52	84 (100)	424	9	433 (100)		32	7	39 (100)
Viral load at early response (2–6 weeks), IU/mL														
<15		294	41	335 (74)	20	33	53 (76)	250	4	254 (73)		24	4	28 (78)
≥15 and <1,000		93	18	111 (24)	6	11	17 (24)	82	4	86 (25)		5	3	8 (22)
≥1,000 and <1 million		4	0	4 (1)	0	0	0 (0)	4	0	4 (1)	NS	0	0	0 (0)
≥1 million		4	0	4 (1)	0	0	0 (0)	4	0	4 (1)		0	0	0 (0)
Total		395	59	454 (100)	26	44	70 (100)	340	8	348 (100)		29	7	36 (100)
Viral load at end of treatment (+/-2 weeks), IU/mL														
<15		296	28	324 (98)	13	20	33 (97)	257	5	262 (98)		26	3	29 (100)
≥15 and <1,000		2	0	2 (1)	0	0	0 (0)	2	0	2 (1)		0	0	0 (0)
≥1,000 and <1 million		1	0	1 (0)	1	0	1 (3)	0	0	0 (0)		0	0	0 (0)
≥1 million		4	0	4 (1)	0	0	0 (0)	4	0	4 (2)		0	0	0 (0)
Total		303	28	331 (100)	14	20	34 (100)	263	5	268 (100)		26	3	29 (100)
SVR 10 or more weeks after end of treatment, IU/mL														
<15		370	41	411 (99)	20	27	47 (98)	319	9	328 (99)		31	5	36 (100)
≥15 and <1,000		0	0	0 (0)	0	0	0 (0)	0	0	0 (0)		0	0	0 (0)
≥1,000 and <1 million		3	1	4 (1)	0	1	1 (2)	3	0	3 (1)		0	0	0 (0)
≥1 million		1	0	1 (0)	0	0	0 (0)	1	0	1 (0)		0	0	0 (0)
Total		374	42	416 (100)	20	28	48 (100)	323	9	332 (100)		31	5	36 (100)
Adherent, %		93.50	78.60	91.70	90.60	75.90	81.40	93.50	100	93.60	<.001	96.90	71.40	92.30
Adherence^†^ to 3D	mean (SD)	0.97 (0.13)	0.87 (0.25)	0.96 (0.15)	0.96 (0.14)	0.85 (0.27)	0.89 (0.23)	0.97 (0.13)	1 (0)	0.97 (0.13)		0.99 (0.06)	0.83 (0.29)	0.96 (0.14)
**Ribavirin Users**		**n = 334**	**n = 66**	**n = 400**	**n = 27**	**n = 51**	**n = 78**	**n = 284**	**n = 8**	**n = 292**		**n = 23**	**n = 7**	**n = 30**
Adherence^†^ to Ribavirin	mean (SD)	0.94 (0.15)	0.86 (0.26)	0.93 (0.17)	0.99 (0.05)	0.87 (0.25)	0.91 (0.21)	0.94 (0.15)	0.76 (0.27)	0.93 (0.16)		0.97 (0.12)	0.89 (0.30)	0.95 (0.18)
Adherence^†^ to 3D and Ribavirin	mean (SD)	0.96 (0.11)	0.87 (0.21)	0.94 (0.14)	0.98 (0.05)	0.86 (0.21)	0.90 (0.18)	0.96 (0.12)	0.88 (0.14)	0.95 (0.12)		0.98 (0.07)	0.86 (0.26)	0.95 (0.14)

HCV, hepatitis C virus; SD, standard deviation; SVR, sustained virological response.

* Values are numbers (percentages) unless otherwise indicated.

^†^ Adherences are in percentage of days covered.

Five participants did not achieve a sustained viral response (Table 4): three men and two women, ages 52–80. None of these participants was HIV or HBV positive and none had received a liver transplant (data not shown). Four of the five participants had genotype 1b and were given a 12-week regimen, and the fifth had genotype 1a and was given a 24-week regimen. One participant had been dispensed all prescriptions for the 24-week regimen, had a detectable viral load at the early response assessment, and had a reduction in viral load from 4,330,000 to 24,400 IU/mL. Three other non-responders were dispensed less than 80% of the 3D protocol. The fifth non-responder acquired all of the prescribed medication and had a higher viral load when assessed for SVR (140,000 IU/mL) than at baseline (130,000 IU/mL). This participant had compensated cirrhosis and stage 4 fibrosis, initiated the 3D protocol less than one year after a diagnosis of HCV without having been previously, was a non-smoker, without diabetes or chronic kidney disease, HIV, or HBV co-infection. Laboratory values for this participant prior to treatment initiation were as follows: ALT 62 U/L, AST 88 U/L, platelet count 25 10^9^/L, and APRI 1.8.

There were six deaths among the participants, three male and three female, aged 53–80. Four of the deaths occurred prior to the end of treatment and the remaining two deaths occurred after the end of treatment. None of them were HIV or HBV positive and none had received a liver transplant. Four of these participants had cirrhosis, among whom, three had decompensated cirrhosis. Five of the six had a negative viral load at the early response viral load test, and the sixth died within six weeks of treatment initiation. All of the deaths were reported in accordance with the standard practices of safety and monitoring of CHS.

**Fig 1:**
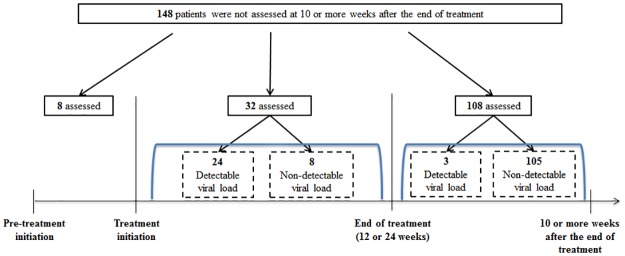
Results of last viral load among participants not assessed for SVR at 10 or more weeks after the end of treatment. Abbreviations: SVR, sustained virological response.

There were 148 participants who were not assessed for SVR (Fig 1). Of those, 108 were tested at least once after the end of treatment and 105 had a non-detectable viral load. Of the 40 who were not tested for a viral load after the end of treatment, 32 were tested at least once after the initiation of treatment, among which the last recorded viral load was non-detectable in 24 participants and detectable in eight participants.

A comparison of those who were and were not assessed for SVR is presented in the appendix material (S1 Table). Baseline demographic characteristics and clinical co-variates were not significantly different between the two groups.

## Discussion

In this large observational study, the 3D protocol demonstrated 98.8% effectiveness in producing a sustained virological response in a population that was inclusive of participants regardless of co-morbidities, age, sex, socioeconomic status, years from first diagnosis, and previous treatment regimen. These findings are consistent with virological response rates in previously reported phase III clinical trials results for 3D protocol in select subpopulations [1–3, 5, 6, 8]. Our most compelling findings relate to the five participants who did not achieve a sustained virological response; despite extensive review of socioeconomic and clinical co-variates, the only common factor in these five participants was that four of them had less than 80% adherence to the prescribed medication regimen.

In addition to the reporting of SVR in the 416 patients assessed, we also analyzed the demographic characteristics and clinical co-variates of the 148 patients who were not assessed. Of those, 108 (73.0%) had a viral load test after the end of treatment, of whom 105 participants (97%) had non-detectable viral loads.

Reviews of DAA regimens such as Guiterriez et al [4] have called for studies that will help guide clinicians in treatment effectiveness pertaining to people with co-morbidities that are excluded from randomized control trials [4, 7, 9, 14, 23–25]. However, there remain few comparable published studies describing the effectiveness of the 3D protocol in clinical practice. Two such studies [13, 17] were conducted using the database of the Veterans Administration, had large cohorts (>1000 participants), and included detailed baseline health information. However, these studies were both conducted in a certain population which is likely to be biased toward a particular risk profile, as indicated by the fact that they are almost universally male and of racial/ethnic minority. Our study included a population-based cohort representing both sexes, a variety of ages, various regions of birth, and included detailed clinical history at baseline as well as viral load testing at multiple points during and after the course of treatment. There is one additional study that compares the 3D protocol to the sofosbuvir/ledipasvir combination [11], however it followed a smaller sample size and had limited claims-based data on co-morbidities.

The strength of our study was that our participants came from a general population, regardless of the number and the nature of co-morbidities, and therefore reflected the use of the 3D protocol in a diverse and representative population. Additional strengths of this study included its condensed enrollment period (less than one year), and its ability to link to an integrated health records database that included demographic characteristics, laboratory values, and co-existing diagnoses.

There were a number of limitations to our study. First, the 148 participants who were not assessed for an SVR may have biased our interpretation into overestimating the effectiveness. However, the baseline demographic characteristics and clinical co-variates in this group were no different than those who were assessed. While we could expect that the three of the 148 participants who had detectable viral loads after the end of treatment would also have detectable viral loads when assessed for an SVR, we did not have reason to believe that the results of those who previously had non-detectable levels would be different from those whose findings we presented. A second limitation was a lack of available documentation of side effects or adverse events of the 3D protocol. While this was of interest, it cannot reliably be captured in an observational study.

In conclusion, participants who initiated treatment for HCV using the 3D protocol achieved near universal SVR, regardless of genotype subtype, treatment duration, diverse demographic characteristics, or various concurrent conditions. A longitudinal follow-up is required to determine whether the SVR persists and if the risk of complications associated with chronic HCV infection will be reduced. Our current study suggests that among these participants in a population-based health care setting, the 3D treatment protocol may have a monumental impact on patient outcomes.

## Supporting information

S1 Table Demographics of Clalit Members initiating 3D treatment, by viral load at 10 or more weeks (not tested versus tested). 3D, dasabuvir/ombitasvir/paritaprevir/ritonavir; y, years; SD, standard deviation; SES, socioeconomic status; WHO, World Health Organization; GBD, global burden of disease; CKD, chronic kidney disease; ACG, adjusted clinical groups; BMI, body mass index; HCV, hepatitis C virus; ALT, alanine transaminase; AST, aspartate aminotransferase; APRI, AST to platelet ratio index score; HIV, human immunodeficiency virus; HBV, hepatitis B virus. * Adherences are in percentage of days covered.
(DOCX)
