# Supplementary material for: Effectiveness of dasabuvir/ombitasvir/paritaprevir/ritonavir for hepatitis C virus in clinical practice: A population-based observational study
Source: PLoS One. 2017 Jul 7;12(7):e0176858. doi: 10.1371/journal.pone.0176858 (PMC5501432; doi:10.1371/journal.pone.0176858)
Supplement: S1 Table — 3D, dasabuvir/ombitasvir/paritaprevir/ritonavir; y, years; SD, standard deviation; SES, socioeconomic status; WHO, World Health Organization; GBD, global burden of disease; CKD, chronic kidney disease; ACG, adjusted clinical groups; BMI, body mass index; HCV, hepatitis C virus; ALT, alanine transaminase; AST, aspartate aminotransferase; APRI, AST to platelet ratio index score; HIV, human immunodeficiency virus; HBV, hepatitis B virus. * Adherences are in percentage of days covered. (DOCX) [file pone.0176858.s001.docx]

**S1 Table. Demographics of Clalit Members initiating 3D treatment, by viral load at 10 or more weeks (not tested versus tested).**

|  | | **Viral load not tested at 10 or more weeks** | | **Viral load tested at 10 or more weeks** | | **p value** |
| --- | --- | --- | --- | --- | --- | --- |
|  |  | **n** | **%** | **n** | **%** |  |
| **Total** |  | **148** | **100%** | **416** | **100%** |  |
| **Demographics** |  |  |  |  |  |  |
| Age, y | mean (SD) | 61.39 (11.99) |  | 61.90 (11.29) |  | 0.641 |
|  | <55 | 38 | 25.7% | 83 | 20.0% |  |
|  | 55-64 | 50 | 33.8% | 150 | 36.1% |  |
|  | ≥65 | 60 | 40.5% | 183 | 44.0% |  |
| Sex |  |  |  |  |  |  |
|  | Male | 87 | 58.8% | 206 | 49.5% |  |
|  |  |  |  |  |  |  |
| Region of Birth by WHO GBD |  |  |  |  |  |  |
|  | Eastern/Central Europe and Central Asia | 89 | 60.1% | 258 | 62.0% |  |
|  | Western Europe/Israel | 38 | 25.7% | 98 | 23.6% |  |
|  | North Africa/Middle East | 18 | 12.2% | 56 | 13.5% |  |
|  | Other | 3 | 2.0% | 4 | 1.0% |  |
| SES |  |  |  |  |  |  |
|  | Low | 64 | 43.2% | 138 | 33.2% |  |
|  | Medium | 58 | 39.2% | 202 | 48.6% |  |
|  | High | 26 | 17.6% | 76 | 18.3% |  |
| **Medical History** |  |  |  |  |  |  |
| Diabetes |  | 51 | 34.5% | 179 | 43.0% |  |
| Duration of diabetes, y | <10 | 35 | 68.6% | 107 | 59.8% |  |
|  | ≥ 10 | 16 | 31.4% | 72 | 40.2% |  |
|  |  |  |  |  |  |  |
| CKD stage |  |  |  |  |  |  |
|  | CKD 1 | 82 | 55.4% | 249 | 59.9% |  |
|  | CKD 2 | 57 | 38.5% | 141 | 33.9% |  |
|  | CKD 3A | 4 | 2.7% | 16 | 3.8% |  |
|  | CKD 3B | 4 | 2.7% | 6 | 1.4% |  |
|  | CKD 4 | 1 | 0.7% | 2 | 0.5% |  |
|  | CKD 5 Non-Dialysis | 0 | 0.0% | 0 | 0.0% |  |
|  | Renal replacement therapy | 0 | 0.0% | 1 | 0.2% |  |
|  | Missing data | 0 | 0.0% | 1 | 0.2% |  |
| ACG categories |  |  |  |  |  |  |
|  | 0-1 | 12 | 8.1% | 22 | 5.3% |  |
|  | 2-3 | 68 | 45.9% | 189 | 45.4% |  |
|  | 4-5 | 68 | 45.9% | 205 | 49.3% |  |
| Charlson with age |  |  |  |  |  |  |
|  | 0 | 14 | 9.5% | 22 | 5.3% |  |
|  | 1 | 23 | 15.5% | 45 | 10.8% |  |
|  | 2+ | 106 | 71.6% | 336 | 80.8% |  |
|  | Missing data | 5 | 3.4% | 13 | 3.1% |  |
| BMI |  |  |  |  |  |  |
|  | Underweight | 1 | 0.7% | 3 | 0.7% |  |
|  | Normal weight | 44 | 29.7% | 120 | 28.8% |  |
|  | Overweight | 58 | 39.2% | 173 | 41.6% |  |
|  | Obese | 45 | 30.4% | 120 | 28.8% |  |
| Smoking |  |  |  |  |  |  |
|  | Non-smoker | 67 | 45.3% | 252 | 60.6% |  |
|  | Former | 25 | 16.9% | 94 | 22.6% |  |
|  | Current | 56 | 37.8% | 70 | 16.8% |  |
| **HCV-specific markers** |  |  |  |  |  |  |
| ALT, IU/L |  |  |  |  |  |  |
|  | mean (SD) | 75.58 (52.79) |  | 83.39 (65.68) |  | 0.192 |
|  | median (range) | 62.50 (40.00 - 97.50) |  | 66.00 (44.00 - 102.00) |  | 0.328 |
|  |  |  |  |  |  |  |
| AST, IU/L |  |  |  |  |  |  |
|  | mean (SD) | 72.58 (41.10) |  | 80.57 (50.05) |  | 0.081 |
|  | median (range) | 63.00 (44.25 - 90.00) |  | 66.00 (48.00 - 98.00) |  | 0.552 |
| Platelets, 10^9^/L |  |  |  |  |  |  |
|  | mean (SD) | 165.56 (71.61) |  | 157.80 (76.06) |  | 0.279 |
|  | median (range) | 153.50 (119.00 - 210.50) |  | 148.50 (101.25 - 206.00) |  | 0.597 |
| APRI score |  |  |  |  |  |  |
|  | mean (SD) | 1.60 (1.30) |  | 2.23 (3.14) |  | 0.001 |
|  | median (range) | 1.27 (0.63 - 1.95) |  | 1.37 (0.76 - 2.44) |  | 0.793 |
| Fibrosis |  |  |  |  |  |  |
|  | 1 | 0 | 0.0% | 1 | 0.2% |  |
|  | 2 | 0 | 0.0% | 0 | 0.0% |  |
|  | 3 | 43 | 29.1% | 123 | 29.6% |  |
|  | 4 | 91 | 61.5% | 207 | 49.8% |  |
|  | Unclear | 0 | 0.0% | 0 | 0.0% |  |
|  | Missing data | 14 | 9.5% | 85 | 20.4% |  |
| Cirrhosis |  |  |  |  |  |  |
|  | Any | 50 | 33.8% | 181 | 43.5% |  |
|  | Compensated | 35 | 70.0% | 131 | 72.4% |  |
|  | Uncompensated | 15 | 30.0% | 50 | 27.6% |  |
| Liver transplant |  | 1 | 0.7% | 3 | 0.7% |  |
|  |  |  |  |  |  |  |
| HIV |  | 3 | 2.0% | 7 | 1.7% |  |
|  |  |  |  |  |  |  |
| HBV |  | 1 | 0.7% | 6 | 1.4% |  |
|  |  |  |  |  |  |  |
| Previous treatment |  | 102 | 68.9% | 306 | 73.6% |  |
|  | PegIF + Ribavirin | 93 | 62.8% | 284 | 68.3% |  |
|  | PegIF + Ribavirin + Boceprevir | 6 | 4.1% | 6 | 1.4% |  |
|  | PegIF + Ribavirin + Telaprevir | 2 | 1.4% | 12 | 2.9% |  |
|  | PegIF | 1 | 0.7% | 4 | 1.0% |  |
|  | None | 46 | 31.1% | 110 | 26.4% |  |
|  |  |  |  |  |  |  |
| HCV duration |  |  |  |  |  |  |
|  | <1 | 18 | 12.2% | 32 | 7.7% |  |
|  | 1-4 | 34 | 23.0% | 65 | 15.6% |  |
|  | 5-10 | 52 | 35.1% | 160 | 38.5% |  |
|  | >10 | 42 | 28.4% | 157 | 37.7% |  |
|  | Missing data | 2 | 1.4% | 2 | 0.5% |  |
| Adherence* to 3D | mean (SD) | 0.88 (0.25) |  | 0.98 (0.09) |  |  |
| **Ribovirin Users** |  | **n = 101** |  | **n = 299** |  |  |
| Adherence* to Ribavirin | mean (SD) | 0.87 (0.26) |  | 0.95 (0.15) |  |  |
| Adherence* to Ribavirin and 3D | mean (SD) | 0.87 (0.21) |  | 0.97 (0.09) |  |  |

3D, dasabuvir/ombitasvir/paritaprevir/ritonavir; y, years; SD, standard deviation; SES, socioeconomic status; WHO, World Health Organization; GBD, global burden of disease; CKD, chronic kidney disease; ACG, adjusted clinical groups; BMI, body mass index; HCV, hepatitis C virus; ALT, alanine transaminase; AST, aspartate aminotransferase; APRI, AST to platelet ratio index score; HIV, human immunodeficiency virus; HBV, hepatitis B virus.

* Adherences are in percentage of days covered.
